# 722 Select Survey of Human Cadaver Skin Allograft Utilization by 21 Senior Academic Burn Surgeons

**DOI:** 10.1093/jbcr/irad045.197

**Published:** 2023-05-15

**Authors:** William Hickerson, Franco Aveau, Narayan Iyer, James Jeng, Victor Joe

**Affiliations:** LSUHSC, Memphis, Tennessee; US Department of Health and Human Services, Washington, DC; US Department of Health and Human Services, Washington, DC; University of California Irvine, Orange, California; UCI Health, Orange, California; LSUHSC, Memphis, Tennessee; US Department of Health and Human Services, Washington, DC; US Department of Health and Human Services, Washington, DC; University of California Irvine, Orange, California; UCI Health, Orange, California; LSUHSC, Memphis, Tennessee; US Department of Health and Human Services, Washington, DC; US Department of Health and Human Services, Washington, DC; University of California Irvine, Orange, California; UCI Health, Orange, California; LSUHSC, Memphis, Tennessee; US Department of Health and Human Services, Washington, DC; US Department of Health and Human Services, Washington, DC; University of California Irvine, Orange, California; UCI Health, Orange, California; LSUHSC, Memphis, Tennessee; US Department of Health and Human Services, Washington, DC; US Department of Health and Human Services, Washington, DC; University of California Irvine, Orange, California; UCI Health, Orange, California

## Abstract

**Introduction:**

As of 2022, cryopreserved human cadaver skin allograft remains the gold standard by which all other skin substitutes used for burn care are compared. The 2015 Taiwan Color Dust Explosion burn mass casualty engendered the transfer of emergency tranches of skin allograft from reserves in both the US and EU to Taiwan. HHS/ASPR/BARDA/CBRN led an after-action fact-finding mission to Taiwan and concluded that skin allograft would still play a central role as a safety net in US national burn care preparedness, even as BARDA invested in the development of other burn medical countermeasures that would be part of the preparedness strategy. However, before investments in a skin allograft based national strategy could be built, it was critical that the leaders of the Disaster Committee and Organization and Delivery of Burn Care (ODBC) Committee of the American Burn Association (ABA) commission a survey to verify current practice patterns of senior academic burn surgeons on the use of skin allograft.

**Methods:**

The committee chairs curated a list of 21 leading academic burn surgeons in North America. These surgeons’ credentials included having been an ABA president, a member of the ABA Board of Trustees, or having chaired a standing committee of the ABA. All 21 surgeons were actively practicing burn care daily. The survey contained 18 questions and was intentionally kept brief (Survey to be provided as Appendix).

**Results:**

Of the 21 surgeons selected, there was 100% participation and completion of the survey. Most were in practice >15 years (62%) and manage >200 patients surgically per year (71%). The predominant use of allograft is to temporize the wound bed when there are inadequate donor sites (48% “Almost Always”). It is “Often” (48%) used to temporize wound beds prior to autografting. However, most are looking for alternatives to allograft (43% “Sometimes”, 43% “Often”). With ongoing development of skin substitutes, most indicated their allograft use will likely decrease, with only 19% indicating “Probably Not”.

**Conclusions:**

Human skin allograft continues to play a central role in surgical burn care management. For the foreseeable future, new technologies are not likely to displace allograft use but rather blend with its use. This brief, select survey is not powered but provides a snapshot of the current role of skin allograft. In the future, we may conduct a broader survey to obtain a validated and more granular view of the use of allograft in modern burn care.

**Applicability of Research to Practice:**

Armed with this information, the US Federal Government acted to increase preparedness after a burn mass casualty. In February 2022, a Request for Proposals for a large vendor-managed inventory of human cryopreserved skin allograft was answered by two separate tissue banks. As of September 2022, $38M was awarded to two partners for procurement and expansion of a vendor managed inventory of cryopreserved human skin allograft to ensure preparedness for rapid response to a burn mass casualty incident.

