# Correction to: 723 Provincial Burn Mass Casualty Incident Planning: Preliminary Results

**DOI:** 10.1093/jbcr/irad133

**Published:** 2023-09-20

This is a correction to: Kathryn King-Shier, RN, PhD, FESC and others, 723 Provincial Burn Mass Casualty Incident Planning: Preliminary Results, *Journal of Burn Care & Research*, Volume 44, Issue Supplement_2, May/June 2023, Page S140, https://doi.org/10.1093/jbcr/irad045.197

In the originally published version of this manuscript, the incorrect author information was provided. The correct order of the authors of this abstract is as follows: Danielle Fuchko, RN BN, Kathryn King-Shier, RN PhD, Vincent Gabriel, MD FRCPC

This error has been corrected online.

